# MOR promotes epithelial-mesenchymal transition and proliferation via PI3K/AKT signaling pathway in human colorectal cancer: MOR promotes EMT and proliferation

**DOI:** 10.3724/abbs.2022114

**Published:** 2022-08-18

**Authors:** Lingling Gao, Li Yang, Yiping He, Yi Liu, Pinbo Xu, Jun Zhang, Sailin Dai, Xing Luo, Zhirong Sun

**Affiliations:** 1 Department of Anesthesiology Fudan University Shanghai Cancer Center; Department of Oncology Shanghai Medical College Fudan University Shanghai 200032 China; 2 Department of Endoscopy Fudan University Shanghai Cancer Center; Department of Oncology Shanghai Medical College Fudan University Shanghai 200032 China

**Keywords:** colorectal cancer, mu-opioid receptor, epithelial-mesenchymal transition, PI3K/AKT signaling pathway, prognosis

## Abstract

The mu-opioid receptor (MOR), a membrane-bound G protein-coupled receptor, is implicated in progression and long-term outcome of several types of tumors. However, the expression and clinical significance of MOR in colorectal cancer (CRC) remain unclear. In this study, a total of 180 paraffin-embedded samples of paired tumors and normal tissues from CRC patients are used to explore expression levels of MOR by immunohistochemistry (IHC). Results show that MOR is highly expressed in tumors compared with that in paired normal tissues ( *P*<0.0001). MOR expression levels are associated with the degree of differentiation ( *P*<0.001) and the regional lymph node metastasis ( *P*<0.001). In addition, a significant difference is also found in the overall survival (OS) between MOR low- and high-expression groups ( *P*=0.002), especially in patients with TNM stage III or IV CRC ( *P*=0.007). Both univariate ( *P*=0.002) and multivariate ( *P*=0.013) analyses indicated that MOR is an independent risk factor associated with CRC prognosis. We further investigate the mechanism in MOR-positive CRC cell line HCT116. The results show that silencing of *MOR* significantly suppresses epithelial-mesenchymal transition (EMT), in addition to suppressing cell proliferation, migration, and invasion. In addition, the expression of downstream p-AKT is also significantly downregulated, and the above suppression effect could be rescued by PI3K/AKT signaling agonist. We conclude that MOR mediates EMT via PI3K/AKT signaling, facilitating lymph node metastasis and resulting in poor survival of CRC patients. Our findings suggest that MOR is a novel prognostic indicator and the application of opioid receptor antagonists may be a novel therapeutic strategy for CRC patients with high MOR expression.

## Introduction

Over the past decades, considerably increased attention has been paid to the role of the anesthetic technique, analgesia, and other associated perioperative events in the recurrence and metastatic rate of malignancies [ 1– 4] . Retrospective clinical studies have demonstrated a diminished incidence of cancer recurrence following regional anesthesia with lower doses of opioids following surgery for breast, colon, ovarian, and prostate cancers [ 5– 8] . Opioids may promote tumor progression and reduce survival [ 9, 10] . Therefore, an increasing number of studies have been carried out to investigate opioid receptors and their roles in tumor progression and metastasis.

Colorectal cancer (CRC) is a frequently diagnosed malignant cancer and a leading cause of cancer-related death, making it an increasingly severe global problem worldwide [ 11, 12] . Currently, CRC treatment is mainly based on the surgical removal of cancer tissues. During perioperative periods, opioids are routinely given for analgesia. Previous studies of CRC indicated that opioids could affect intestinal epithelium integrity, change the microbiome composition, and modulate the immune system. However, only a few studies addressed the direct effects of opioids on CRC cells [ 13, 14] .

The mu-opioid receptor (MOR), a membrane-bound G protein-coupled receptor, is the main target for most opioids used clinically [15], such as morphine, fentanyl, and heroin [16]. MOR is widely expressed in the central nervous system and peripheral tissue. Previous studies indicated that MOR is highly expressed in various types of human cancers and associated with poor clinical outcomes [ 17– 19] . For example, a previous study about hepatocellular carcinoma (HCC) revealed that high expression of MOR in HCC is associated with metastasis and poor prognosis through mediation of epithelial-mesenchymal transition (EMT) [17], a key step in malignant tumor progression and metastasis. Furthermore, overexpression of MOR was found in human lung carcinoma and assumed to play essential roles in carcinogenesis and cancer progression [ 18, 19] . Silencing of *MOR* was reported to decrease tumor proliferation and metastasis. MOR was also found in CRC cell lines HT-29 and tumor tissue [20]. However, little is known about the difference in its expression level in paired tumors and normal tissues. Furthermore, the relationship between the expression level of MOR and CRC prognosis still awaits further exploration.

In the present study, using paired CRC specimens, for the first time we revealed the relationship between MOR expression level and CRC clinic prognostic. Furthermore, we investigated the mechanism in MOR-positive CRC cell line HCT116. The results showed that silencing of *MOR* significantly suppressed EMT, cell proliferation, migration, and invasion. In addition, the expression of downstream p-AKT was also significantly downregulated, and the above suppression effect could be rescued by PI3K/AKT signaling agonist SC79. This study not only elucidated the possible effect of MOR in CRC progression, but also provided a potential therapeutic strategy for MOR-positive CRC patients.

## Materials and Methods

### Patients and follow-up

A total of 180 patients with colorectal cancer (CRC) who had received surgical treatment at Fudan University Shanghai Cancer Center (Shanghai, China) from April 2008 to October 2009 were enrolled in this study. Tumors and paired normal tissues were collected from the patients and formalin-fixed and paraffin-embedded. Clinicopathological data were retrieved from our prospectively constructed CRC database. The tumor stage was determined according to the seventh edition of the International Union Against Cancer (UICC)/American Joint Committee on Cancer (AJCC) TNM classification. The patients were followed every three months in the first year after surgery. The overall survival (OS) was calculated from the day of surgery to the date of death from CRC or last follow-up. Ethical approval was obtained from the Clinical Research Ethics Committee of Fudan University Shang Cancer Center (Shanghai, China). Signed informed consent was obtained from each patient.

### Cell lines and drugs

The human CRC cell lines HCT116, Caco-2, SW480, and LoVo were all from the Institute of Biochemistry and Cell Biology (Shanghai, China) and maintained in a humidified atmosphere with 5% CO _2_ at 37°C. HCT116, SW480 and LoVo cells were cultured in RPMI-1640 medium (Gibco, Carlsbad, USA) supplemented with 10% fetal bovine serum (FBS; Gibco). Caco-2 cells were cultured in MEM medium (Gibco) supplemented with 10% FBS. SC79, a selective PI3K/AKT signaling pathway agonist, was purchased from Cell Signaling Technology (CST; Beverly, USA). SC79 was dissolved in steriledimethyl sulfoxide to make a 10 mM stock solution. All stock solutions were stored in aliquots at –20°C and diluted to make working solutions before use.

### Silencing of *MOR*

To construct a *MOR*-knockdown lentivirus, target sequences were designed as follows: sh-MOR-i1: 5′-CACGAACGCCAGCAATTGCACTGAT-3′, and shMOR-i2: 5′-ACTGATGCCTTGGCGTACTCAAGTT-3′. Short hairpin Mu Opioid Receptor (MOR) plasmid (sh-MOR; GeneChem, Shanghai, China), or short hairpin negative control plasmid (sh-NC: 5′-TTCTCCGAACGTGTCACGT-3′; GeneChem) were transfected into cells using Lipofectamine™ 2000 Transfection Reagent (Thermo Fisher, Waltham, USA) for 24 h. Positive cells were selected at 2 weeks following puromycin treatment. The expression of MOR was identified by real-time PCR.

### Real-time PCR

Total RNA from cell lines was extracted using an RNA isolation kit (QIAGEN, Hilden, Germany). RNA was subject to cDNA synthesis with a PrimeScript RT reagent kit (TaKaRa, Dalian, China). The cDNA was used as the template for real-time PCR analysis on an ABI 7200 analyzer (Applied Biosystems, Foster City, USA) with the fluorescent probe SYBR Green I (Tiangen, Beijing, China). Relative expression levels of the genes were normalized to the housekeeping gene *GAPDH*. Each experiment was independently repeated at least 4 times.

### Tissue microarray construction

Tissue microarrays (TMA) were constructed as previously described [21]. The representative regions of each tissue were first singled out of hematoxylin and eosin-stained sections and marked on the individual paraffin blocks. At least two tissue cores were acquired from each specimen, measuring 1.8 mm in diameter and 1.0–3.0 mm in length depending on the depths of tissue in the donor block. Each core was precisely arrayed into a new paraffin block. These microarrays were serially sectioned (4 μm) and stained with hematoxylin and eosin to ensure tissue sampling and completeness. The unstained sections were baked overnight at 56°C in preparation for immunohistochemistry staining.

### Immunohistochemistry staining

Immunohistochemistry staining of the chip was performed using a two-step protocol (Novolink Polymer Detection System; Novocastra, Newcastle, UK). Briefly, sections were deparaffinized with xylene and rehydrated with graded ethanol solutions. After the endogenous peroxide activity in the slides was blocked by incubation in 0.3% H _2_O _2_, retrieval was performed by placing the sections in boiling citrate or ethylenediamine tetraacetic acid (EDTA) buffer for 10 min, and nonspecific binding sites were blocked with 5% goat serum (Beyotime, Shanghai, China) in PBS. The slides were incubated with a primary antibody against MOR (1:1000; Abcam, Cambridge, USA) and then with horseradish peroxidase (HRP)-conjugated secondary antibodies (1:400; KPL, Gaithersburg, USA), followed by incubation with 3′-diaminobenzidine (DAB) solution (Sigma-Aldrich, St Louis, USA). Slides without primary antibodies were included as negative controls in all assays.

All specimens were counterstained with hematoxylin and examined under a light microscope (TCS-SP5; Leica Camera AG, Hamburge, Germany). Staining intensity was referred to as the standard of results judgment. The intensity of peroxidase deposits, ranging from light beige to dark brown, was assessed visually in the tumor cell membrane and scored as 0 (negative), 1 (weak), 2 (moderate), or 3 (strong). For statistical analysis, a score of 0 was considered negative expression, scores of 1–3 were considered positive expression, scores of 0–1 were considered low expression, and scores of 3–4 were considered high expression. Two pathologists in gastroenterology who were blinded to the clinical data pathologically assessed all specimens independently.

### Cell counting kit-8 assay

HCT116 cells were plated into 96-well plates at 2×10 ^4^ cells/well and cultured for 0, 24, 48, and 72 h. Then, 100 μL medium, including 10 μL cell counting kit-8 (CCK8; Dojindo, Tokyo, Japan), was added to each well. After incubation for 1 h, the absorbance value (OD value) was detected at 450 nm with a microplate reader.

### Cell migration and invasion assays

Six-well plates with 8-μm pore size inserts (Corning, New York, USA) were used to assess cell migration (without Matrigel) or invasion (with Matrigel). Briefly, 1×10 ^5^ HCT116 cells were seeded in the upper chamber, and 600 μL medium with 30% FBS was added to the lower chamber and cultured for 48 h. Then, the migrated or invaded cells were fixed with 4% paraformaldehyde for 1 h and then stained with 0.1% crystal violet solution for 30 min. Five fields were randomly selected to take photos and calculate the cell numbers of migration and invasion.

### Western blot analysis

Cells were lysed and protein concentration was quantified using a protein assay kit (Beyotime). Then, protein samples were separated by SDS-PAGE and transferred to a polyvinylidene fluoride (PVDF) membrane. After being blocked with 5% milk at room temperature for 1 h, the membrane was incubated overnight at 4°C with the following antibodies as indicated: MOR (ab134054; Abcam), AKT (4685S; CST), phospho-AKT (4060S; CST), β-catenin (ab16051; Abcam), N-cadherin (ab18203; Abcam), Twist (ab18203; Abcam), E-cadherin (ab76055; Abcam) and β-actin (4970s; CST). Then, HRP-conjugated secondary antibody (KPL) was added and incubated at room temperature for 2 h. Finally, protein bands were visualized using ECL system (Thermo Fisher), and quantified with a chemiluminescence gel imaging system (Tanon 5200; Tanon, Beijing China).

### Statistical analysis

Associations between MOR expression and clinicopathological characteristics of CRC were assessed with the χ2 tests. A log-rank test was constructed for overall survival analysis. Univariate and multivariate analyses were based on the Cox proportional hazards regression model. Two-tailed Student’s *t*-test was used to assess statistical significance between conditions. *P*<0.05 was considered to have statistical significance. All statistical analyses were performed using SPSS for Windows (Version13.0; SPSS Inc., Chicago, USA).

## Results

### Overexpression of MOR in CRC tumor tissues

**Figure 1:**
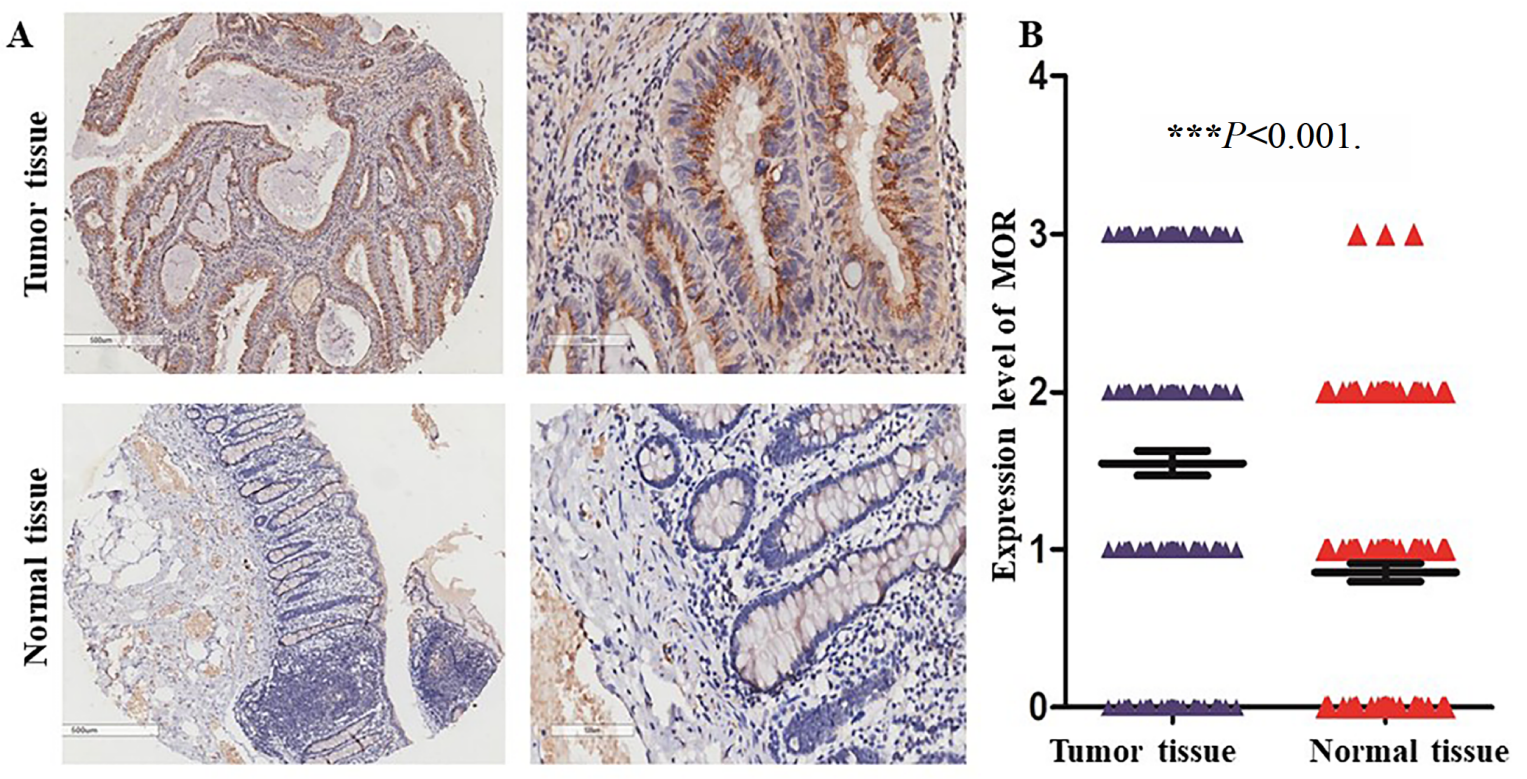
Figure 1 Overexpression of MOR in CRC tumor tissues (A) Representative images of MOR expression of paired normal and tumor tissues (original magnification: × 40 for the left two images, and × 200 for the right two images). (B) Based on the expression intensity of MOR, 180 patients were divided into 4 groups: strong (score=3), moderate (score=2), weak (score=1) and negative (score=0). *** P<0.001.

The clinical and pathological characteristics of the 180 patients with CRC were summarized in Table 1. Paired normal and tumor tissues were collected from each patient and stained by IHC using anti-MOR antibody. As shown in Figure 1, MOR was mainly expressed in the cytoplasm of CRC cells ( Figure 1A). Compared with that in normal tissues, the expression level of MOR was significantly higher in the paired tumor tissues ( Figure 1B; *P*<0.0001). According to the scoring criteria described in the methods section, the positive expression rate of tumor tissue and normal tissue was 80.6% (145/180) and 63.9% (115/180), respectively, and the high expression rate was 52.8% (95/180) and 20% (36/180) respectively.

**Table:** **Table 1** Clinical characteristics of the 180 colorectal cancer patients

Variables	Number	Percentage (%)
Age (years)		
<60	131	72.8
≥60	49	27.2
Gender		
Male	99	55.0
Female	81	45.0
Tumor size (cm)		
<5	109	60.6
≥5	71	39.4
Gross appearance		
Exophytic	55	30.6
Ulcerative	20	11.1
Infiltration	105	58.3
Tumor differentiation		
I–II	141	78.3
III–IV	39	21.7
Depth of invasion		
T1–T2	15	8.3
T3–T4	165	91.7
Lymph node metastasis		
Negative	106	58.9
Positive	74	41.1
Liver metastasis		
Negative	166	92.2
Positive	14	7.8
Stage		
I–II	104	57.8
III–IV	76	42.2

### Association of MOR expression with clinicopathological characteristics

To better understand the clinical significance of MOR expression in CRC, we correlated MOR expression level with a series of clinicopathological parameters ( Table 2). According to the expression intensity of MOR in tumor tissues, 180 patients were classified into the MOR high-expression group ( *n*=95) and MOR low-expression group ( *n*=85). The data showed that MOR expression was significantly correlated with the degree of differentiation ( *P*<0.001) and regional lymph node metastasis ( *P*<0.001). There was no significant correlation of MOR expression with the patients’ age, gender, infiltration depth, primary tumor parameters, distant metastasis, or TNM stage grouping.

**Table:** **Table 2** The relationship between the clinical characteristics and MOR expression level in 180 patients with colorectal cancer

Variables	Score=0/1 ( *n*=85)	Score=2/3 ( *n*=95)	*P* value
Age (years)			
<60	22 (12.2)	27 (15.0)	0.702
≥60	63 (35.0)	68 (37.8)	
Gender			
Male	42 (23.3)	57 (31.7)	0.154
Female	43 (23.9)	38 (21.1)	
Stage			
I–II	55 (30.6)	49 (27.2)	0.075
III–IV	30 (16.7)	46 (25.7)	
Tumor size (cm)			
<3	5 (2.80)	5 (2.80)	0.382
3–5	33 (18.3)	28 (15.6)	
>5	47 (26.1)	62 (34.4)	
Gross appearance			
Exophytic	30 (16.7)	25 (13.9)	0.076
Ulcerative	5 (2.80)	15 (8.30)	
Infiltration	50 (27.8)	55 (30.5)	
Histological type			
Adenocarcinoma	75 (41.7)	83 (46.1)	0.859
Mucinous adenocarcinoma	10 (5.60)	12 (6.60)	
Tumor differentiation	78 (43.3)	63 (35.0)	<0.001
I–II (Well)	7 (3.9)	32 (17.8)	
III–IV (Poor)			
Depth of invasion			
T1–T2	6 (3.30)	9 (5.00)	
T3–T4	79 (43.9)	86 (47.8)	0.558
Lymph node metastasis			
T0	60 (33.3)	46 (25.6)	<0.001
T1	23 (12.7)	30 (16.7)	
T2	2 (1.10)	19 (10.6)	
Metastasis			
Negative	80 (44.4)	86 (47.8)	0.369
Positive	5 (2.80)	9 (5.00)	

### High expression level of MOR is associated with poor overall survival of CRC patients

**Figure 2:**
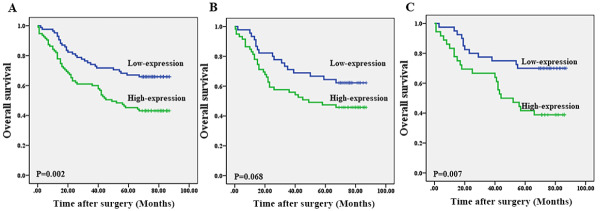
Figure 2 Strong MOR expression correlates with poorer overall survival (A) The OS in the MOR low-expression group was higher than that in the MOR high-expression group. (B,C) The difference of OS was obvious in TNM stage III–IV patients (C; P=0.007), but not in TNM stages I–II (B; P=0.068)

Furthermore, we used Kaplan–Meier survival analysis to explore the impact of MOR expression on overall survival. As shown in Figure 2, there was a significant difference in overall survival (OS) between the patients of the MOR high-expression group and the patients of the MOR low-expression group ( Figure 2A; *P*=0.002). Next, subgroup analysis showed that the difference of OS was noticeable in TNM stage III–IV patients ( Figure 2B; *P*=0.007) but not in TNM stage I–II patients ( Figure 2C; *P*=0.068).

### MOR is an independent prognostic risk factor for CRC

The above results indicated that MOR is over-expressed in CRC tissue and correlated with poor OS in CRC patients. Finally, univariate and multivariate analyses based on the Cox proportional hazards regression model were used to analyze the relationship between MOR expression and the clinical characteristics, and the results indicated that MOR is a potential independent prognostic risk factor for CRC ( Table 3).

**Table:** **Table 3** Univariate and multivariate analyses of prognostic factors

Variables	Number( *n*=180)	Univariate *P*	Multivariate analysis
HR (95% CI)	*P*
Age (year) (<60 vs ≥60)	131/49	0.061		
Gender (male/female)	99/81	0.213		
Tumor size (cm) (<5 vs ≥5)	109/71	0.015	1.676 (1.030–2.725)	0.038
Gross appearance (exophytic vs ulcerative vs infiltration)	55/20/105	0.926		
Histological type (mucinous adenocarcinoma vs adenocarcinoma)	22/158	0.859		
Tumor differentiation (III+IV vs I+II)	39/141	0.551		
T (T1+T2 vs T3+T4)	15/165	0.104		
Metastasis (absent vs present)	166/14	<0.001	4.812 (2.103–11.007)	<0.001
MOR expression (Score 0–1 vs Score 2–3)	85/95		1.832 (1.139–2.947)	0.013

### Role of MOR in the proliferation, migration and invasion of CRC cells

**Figure 3:**
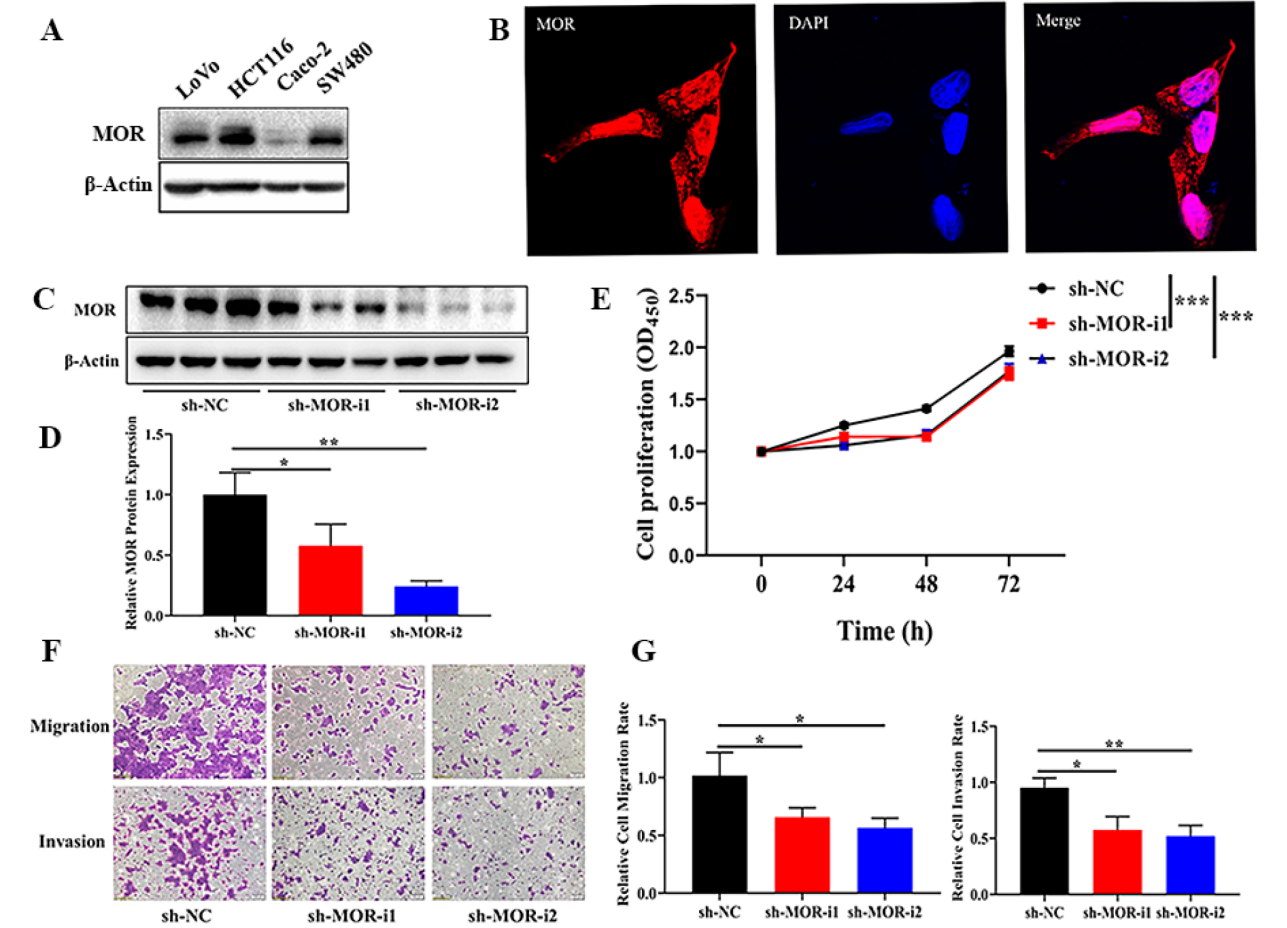
Figure 3 Role of MOR in CRC proliferation, migration, and invasion (A) Western blot analysis was used to detect the MOR expression in four CRC cell lines. (B) Fluorescence microscopy was used to detect the expression of MOR in HCT116 (Red, MOR; Blue, DAPI; Magnification: ×400). (C)Representative western blots showing the knockdown of MOR in HCT116 cells by transient transfection with sh-MOR. sh-NC was used as control. (D) The relative protein level was shown. (E) Cell proliferation assay and the statistical results. (F,G) Migration and invasion assay and statistical results. * P<0.05, ** P<0.01, and *** P<0.001.

Western blot analysis was used to examine the expression of MOR in four CRC cell lines. It was found that MOR was strongly expressed in the HCT116 cell line, whereas moderate signals were observed in LoVo and SW480 cell lines, and the Caco-2 cell line almost did not express MOR ( Figure 3A). Therefore, MOR-high expression cell line HCT116 was chosen for further study. As shown in Figure 3B, MOR was mainly expressed in the cell cytoplasm.

We next examined whether MOR is necessary for the tumorigenic phenotypes of HCT116 cell by downregulating MOR expression with siRNA. The introduction of MOR-specific siRNA (sh-MOR) into HCT116 cells significantly reduced the expression level of MOR relative to control cells transfected with scrambled siRNA (sh-NC) ( Figure 3C,D). MOR knockdown dramatically suppressed their proliferation, migration, and invasion compared with that of their corresponding control group ( Figure 3E–G). To further demonstrate the role of MOR in CRC cells, we repeated the experiment with another cell line and similar trend was found in the LoVo cell line ( Supplementary Figure S1).

### Impact of *MOR* silencing on EMT in CRC cells

**Figure 4:**
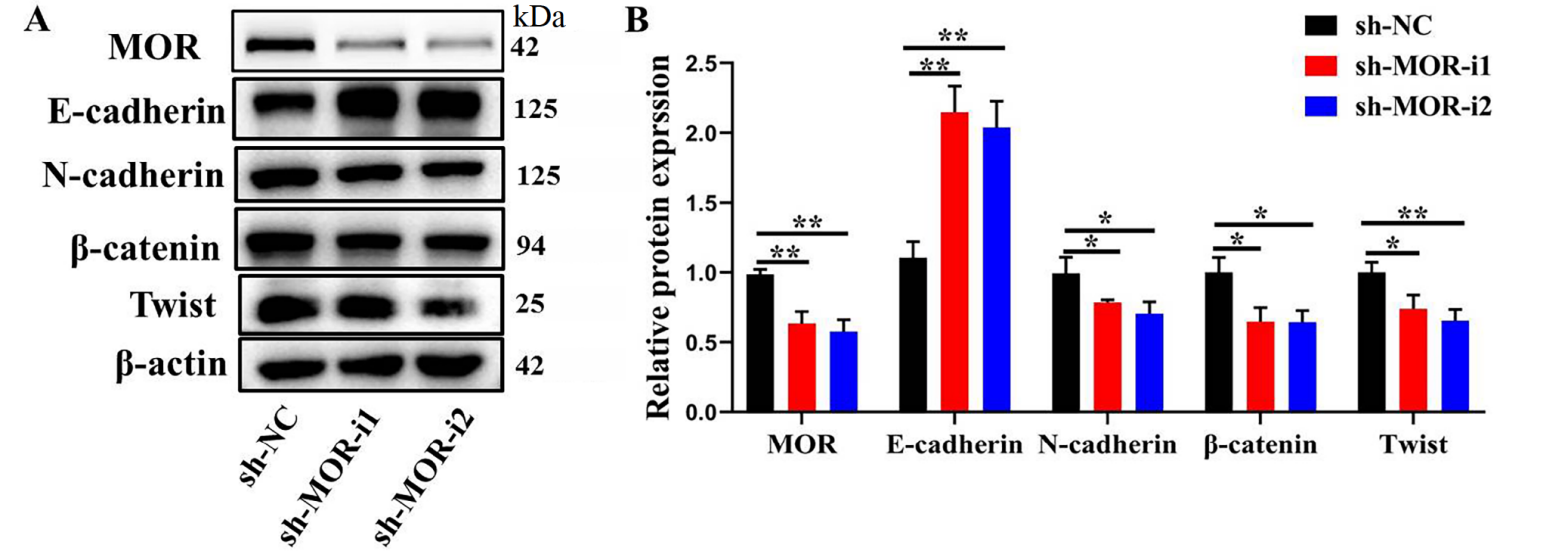
Figure 4 Impact of *MOR* silencing on epithelial-mesenchymal transition in CRC cells (A) Representative western blots showing the expressionsof EMT-markers after transfection with sh-MOR in CRC cells. (B) The relative expression level was shown. * P<0.05 and ** P<0.01.

Because EMT has been demonstrated as a key step in initiating CRC migration and invasion, and silencing of *MOR* is correlated with suppressed cell metastatic abilities of CRC cells, we next examined the impact of *MOR* silencing on EMT. In shMOR-transfected HCT116 cells, downregulation of β-catenin, N-cadherin, and Twist expressions and upregulation of E-cadherin expression were observed ( Figure 4).

### Impact of PI3K/AKT pathway agonist on migration, invasion and proliferation in *MOR*-silenced CRC cells

**Figure 5:**
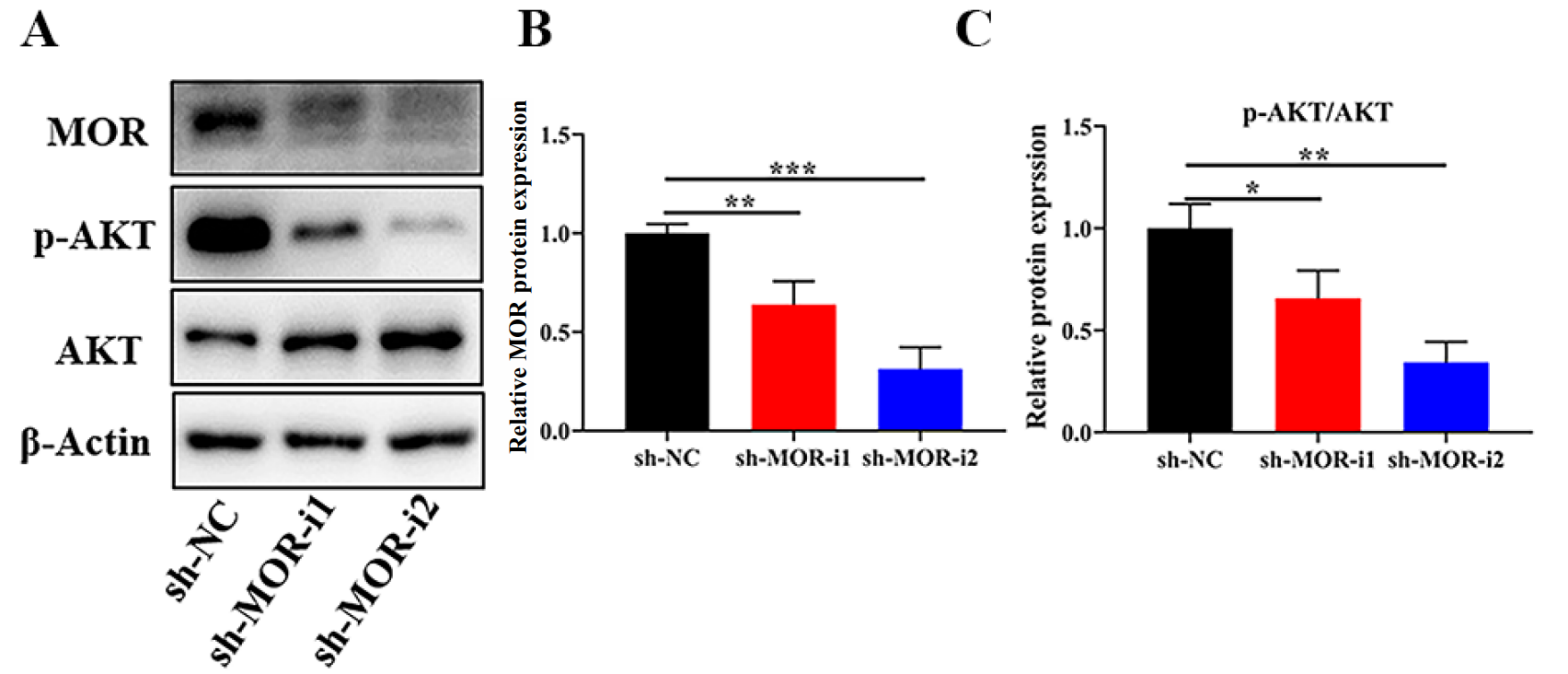
Figure 5 Impact of *MOR* silencing on PI3K/AKT pathways (A) Representative western blots showing the expressions of MOR and p-AKT after transfection with sh-MOR in CRC cells. (B,C) The relative expression level was shown. * P<0.05, ** P<0.01, and *** P<0.001.

**Figure 6:**
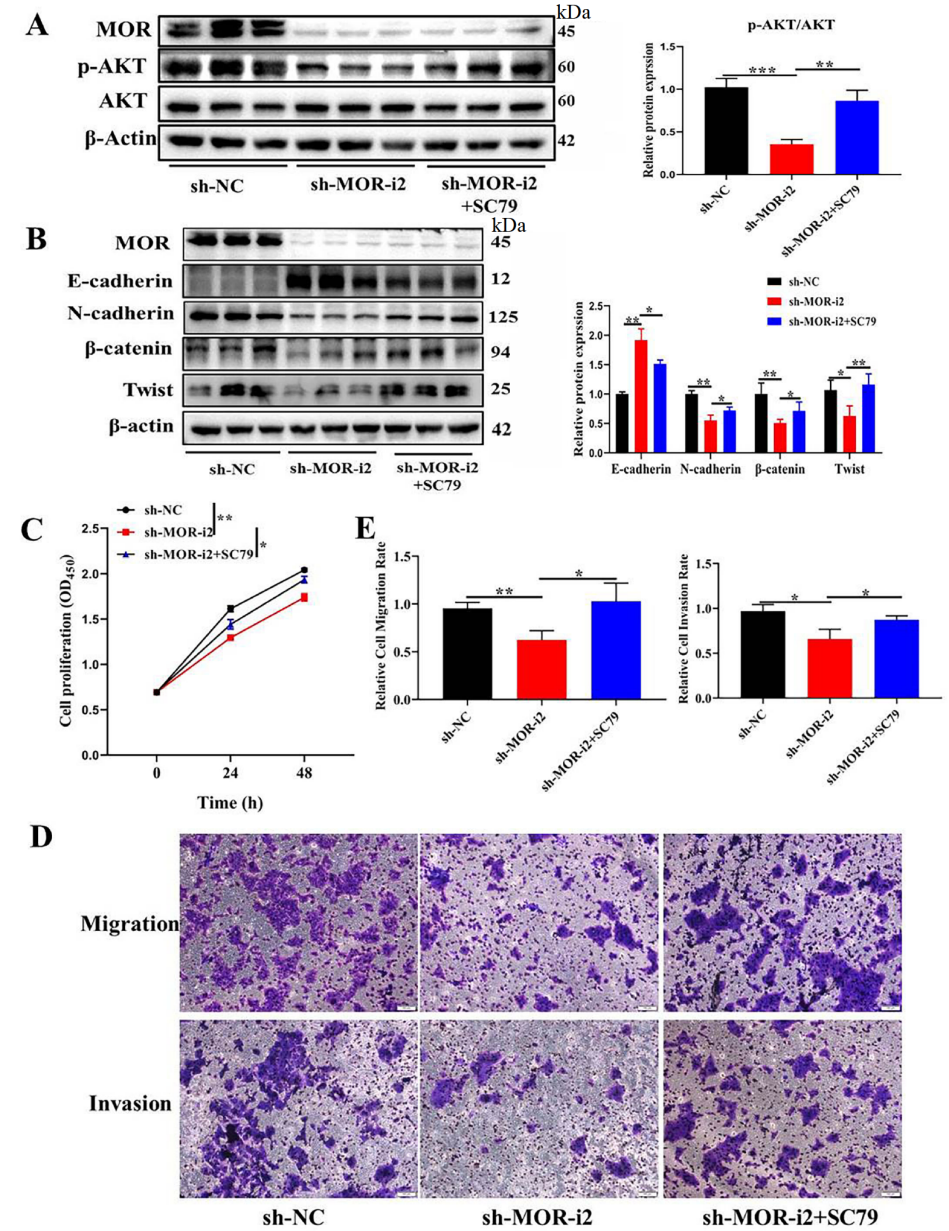
Figure 6 Impact of PI3K/AKT pathway agonist on EMT and proliferation in *MOR*-silenced CRC cells (A) Representative western blots showing the expressions of MOR and p-AKT after transfection with sh-MOR or/and treatment with SC79PI3K/AKT pathways agonist in CRC cells. (B) Representative western blots showing the expressions of EMT-markers. (C) Cell proliferation assay and the statistical results. (D,E) Migration and invasion assay and statistical results. * P<0.05 and ** P<0.01.

Previous studies suggested that MOR activation could lead to the activation of downstream PI3K pathways. We next detected the expression level of p-AKT in sh-MOR cells. As shown in Figure 5, compared with that in the sh-NC group, p-AKT was significantly downregulated in the sh-MOR group. We further explored the impact of the PI3K/AKT pathway agonist on CRC progression. Firstly, after *MOR-*silenced cells were treated with PI3K/AKT pathway agonist, significant upregulation of p-AKT was detected ( Figure 6A).We then explored the EMT markers. As shown in Figure 6B, the mesenchymal markers β-catenin, N-cadherin, and Twist were significantly downregulated, while epithelial marker E-cadherin was upregulated by *MOR* silencing. But the above function could be rescued by PI3K/AKT pathway agonist. We then explored the tumorigenic effect. Cell proliferation ( Figure 6C), migration, and invasion ( Figure 6D,E) abilities were significantly enhanced by treating *MOR*-silenced cells with PI3K/AKT pathway agonist SC79.These results indicate that activation of MOR downstream pathway PI3K/AKT contributes to the EMT of tumor cells.

## Discussion

The choice of anesthetic techniques and other perioperative factors is increasingly implicated in long-term patient outcomes after cancer surgery [ 22– 25] . Opioids have received widespread attention as the most widely used drugs for cancer patients with both acute and chronic pain and during the perioperative period. Several retrospective studies suggested that high opioid dosage was associated with poor clinical prognosis in various tumors, and there is increasing awareness of the value of opioid-sparing techniques [ 15, 26, 27] . The opioid system, consisting of three G protein-coupled receptors—mu, delta, and kappa (MOR, DOR, and KOR, respectively)—has been identified mainly in the central nervous system and also occurs in a wide spectrum of peripheral organs and tissues [28]. Among opioid system subtypes, MOR is the main therapeutic target for most clinically used opioids such as morphine, fentanyl, and heroin, with binding affinity nearly two orders of magnitude greater than that of the other two types of opioid receptors [29]. The expression of MOR has been found to be upregulated in several types of cancers [ 17, 30] . MOR was also found to be expressed in CRC tissue and cell lines [20]. However, to our knowledge, the specific expression differences, compared with those of normal tissue, are still unknown. Our study is the first attempt using tissue microarrays to verify that MOR expression level is significantly higher in tumor tissue than in paired normal tissue.

Apart from the high expression level of MOR in cancer cells, the relationship between MOR expression level and cancer prognosis is still under debate. For instance, Chen *et al*. [17] revealed that a high expression level of MOR was correlated with aggressive clinicopathological features and a worse prognosis of liver cancer, but a previous study from esophageal squamous cell carcinoma showed no statistical significant difference between MOR expression status and overall survival [31]. We found that MOR expression level was significantly correlated with tumor differentiation and regional lymph node metastasis. So we confirm that MOR plays an important role in CRC. More importantly, our univariate and multivariate analyses showed that high expression of MOR is an independent prognostic factor for OS. Patients with TNM stage III or IV CRC and high expression of MOR had worse OS than those in the low expression group. On the other hand, MOR expression level had no effect on OS in TNM stage I or II patients. The current study demonstrated for the first time that high expression level of MOR is associated with poor CRC prognosis.

It is well known that various clinical parameters are related to CRC prognosis, such as tumor size, lymph node status, and TNM stage. The above parameters reflect the frequency of advanced disease stages at diagnosis and predict tumor recurrence and CRC progression. The current study suggests that MOR expression level may be a potential new biomarker of CRC that can be used for predicting patient prognosis, which may have a significant impact on improving therapeutic effects for patients with advanced CRC.

It is also well known that MOR activation can lead to the activation of downstream MAPK and PI3K pathways [ 32, 33] . To understand the signaling cascades of MOR signaling-induced cancer progression, many preclinical experiments have been done, which also demonstrated that the above two pathways are also involved in the regulation of tumor progression. For example, morphine in clinically relevant doses promotes tumor neovascularization in a human breast tumor by activating the survival signal PI3K/AKT, inhibiting apoptosis, and promoting cell cycle progression through increasing cyclinD1 [26]. MOR was also proven to facilitate the progress of EMT, which ultimately leads to greater metastatic potential [ 17, 34] . The above evidence strongly suggests a significant regulatory role of MOR at the molecular level, but this regulatory effect in CRC remains poorly understood.

Although our data indicated that high expression of MOR is associated with lymph node metastasis and prognosis of CRC, the mechanism and downstream mediators remain incompletely defined. Therefore, we further performed *in vitro* cell experiments. We found that MOR is strongly expressed in the HCT116 cell line, while moderate signals were observed in LoVo and SW480 cell lines, and the Caco-2 cell line almost did not express MOR. We chose MOR high-expression cell line HCT116for further study. Our results indicated that silencing of *MOR* by siRNA significantly suppressed cell EMT and proliferation. Moreover, the MOR downstream PI3K/AKT pathway was also significantly suppressed in *MOR*-silenced cells. Furthermore, the above suppression effect can be rescued by a PI3K/AKT signaling agonist. These results revealed the key roles of MOR in promoting poor CRC prognosis by promoting EMT via the AKT pathway, which may play a crucial role in CRC development. Further studies will be carried out in the future to elucidate the specific regulatory mechanism of MOR in promoting the CRCEMT process.

In summary, we verified for the first time that MOR is overexpressed in tumor tissue compared with that in paired normal tissue. Its expression level is associated with poor prognosis of CRC, and it is an independent prognostic risk factor for CRC. Overexpression of MOR plays a key oncogenic role in CRC by facilitating CRC cell proliferative and metastatic capacities through the induction of EMT via the AKT signaling pathway. Our data further suggest that MOR agonists used in CRC patients should be carefully evaluated, and application of MOR antagonists may be a potential therapeutic strategy for CRC patients.

## Supplementary Data

Supplementary data is available at *Acta Biochimica et Biophysica Sinica* online.

## Supporting information

8-22314supplementary_Figure_S1
