# Supplementary material for: MOR promotes epithelial-mesenchymal transition and proliferation via PI3K/AKT signaling pathway in human colorectal cancer: MOR promotes EMT and proliferation
Source: Acta Biochim Biophys Sin (Shanghai). 2022 Aug 18;55(1):72–80. doi: 10.3724/abbs.2022114 (PMC10157524; doi:10.3724/abbs.2022114)
Supplement: 8-22314supplementary_Figure_S1 [file 8-22314supplementary_Figure_S1.pdf]

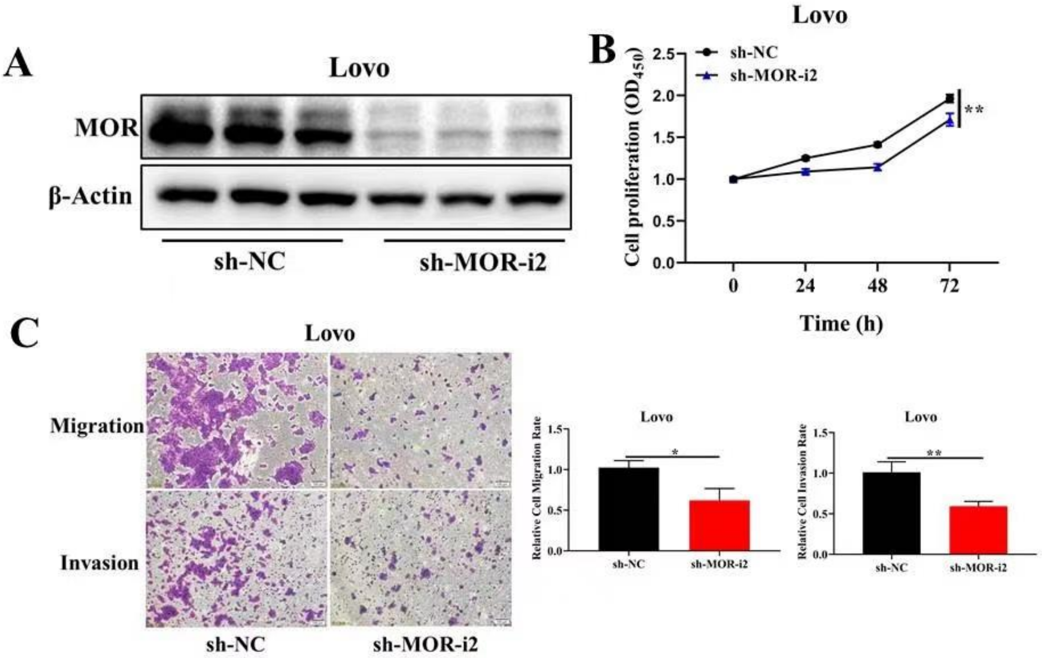

Supplementary Figure S1. Role of MOR in CRC proliferation, migration, and invasion in LoVo cells (A) Representative western blots showing the knockdown of *MOR* in LoVo cells by transient transfection with sh-MOR. (B) Cell proliferation assay and the statistical results. (C) Migration and invasion assay and statistical results. **P*<0.05, ***P*<0.01.
